# All-Time Best Norwegian Track and Field Athletes: To What Extent Did They Achieve Outstanding Results at The Ages of 15 and 18 Years?

**DOI:** 10.3390/ijerph17197142

**Published:** 2020-09-29

**Authors:** Leif Inge Tjelta, Ola Tjensvoll

**Affiliations:** Faculty of Arts and Education, University of Stavanger, 4036 Stavanger, Norway; olatjensvoll@hotmail.com

**Keywords:** elite athletes, athletics, youth performance, talent identification

## Abstract

The aim of the study was to determine how many Norwegian athletes who, during all the times they had achieved the European Athletics Championship 2020—Entry Standards (EAC20ES), were also ranked among the 20 all-time-best athletes at the ages of 15 and 18 years. The number of athletes who achieved the EAC20ES during their career, and the percentage of those who were among top 20 in the age groups 15 and 18 years, were determined from the Norwegian all-time-best results lists. A total of 202 athletes achieved the EAC20ES in the studied time period. Of these, 14.4% and 42.1% were ranked among the top 20 all-time best in one or more events at the ages of 15 and 18 years, respectively. However, among those who had won an international gold medal, these percentages were much higher. Eight out of 12 champions (66.7%) were ranked among the top 20 all-time best in one or more event at 15 years of age, and 11 of 12 champions (91.6%) were ranked among the top 20 all-time best at 18 years of age. Athletes that went on to win international championships typically performed better as adolescents compared to other athletes who also reach an international level as seniors. However, due to the low number of international champions, the date should be interpreted with caution.

## 1. Introduction

Exceptional sporting performance, often defined as winning a medal in an international competition, is dependent on motor and physical development over the lifespan [1]. Today, children often focus on training and competition in sports from an early age, in the hope that this will help them to reach an elite level as adults [2,3,4].

It is a debated topic whether long-term success in elite sports can be predicted from performance in youth competitions [5,6] and to what extent early specialization [7] or early diversification is key for success at senior level [8]. Early specialization has been defined as intensive training or competition in organized sport by prepubescent children (<12 years of age) for more than 8 months per year, with a focus on a single sport to the exclusion of other sports and free play [9].

The early specialization framework [7] postulates that training and development for 10,000 hours from early to middle childhood in the primary sport is necessary to achieve exceptional performance.

According to LaPrade, Agel, Baker, Brenner, Cordasco, Côté, Engebretsen, Feeley, Gould and Hainline [10], there is no evidence that young children will benefit from early sport specialization in the majority of sports. However, in some sports like golf, tennis and gymnastics, early specialization has been considered important for the development of certain skills [11]. Early specialization and intensive training during early adolescence can also result in an increased risk of injuries and a decrease in sport enjoyment [12,13].

The early diversification path recommends a greater involvement in a variety of sports before specializing [13,14]. Evidence has proved that the early diversification path where athletes had been engaged in deliberate play during childhood could lead to elite level of performance in sports [15,16,17,18].

Previous research has examined the relationship between youth and adult performance in different sports [19,20,21,22]. For example, Barreiros, Côté and Fonseca [21] found that only one in three athletes who competed at an international level at pre-junior level (≤16 years) in swimming, volleyball, judo or football, also competed internationally at senior level. Conversely, in a study of Norwegian road cyclists by Svendsen, Tønnesen, Tjelta and Ørn [19], race performance at age 18 was found to be the strongest predictor of success at senior level.

In the sport of track and field athletics, the International Association of Athletics Federations (IAAF) arranges the World Junior Championship every other year, for athletes under the age of 20. Hollings and Hume [23] retrospectively examined a cohort of 137 athletes who were Olympic or World Champions, and previous competitors in the World Junior Championship (WJC). Of these, 55% were former medalists and 80% were top eight finishers in WJC. However, a contrasting picture emerges from the same data analyzed prospectively, following 1054 medalists at World Junior Championships from 1986 to 2004. Of the 1054 junior medalists, only 34% became medalists or finalists in international competitions as seniors, whilst a further 12% competed at international level, but never made it to an international final. As many as 54% never participated in international competitions as senior athletes. In a study where the purpose was to examine the lifetime best performance of World Junior Championship finalists and Olympic Games finalists in athletic events, Foss, Sinex and Chapman [1] found that World Junior Championship finalists achieved their career-best performance at a younger age compared to Olympic Games finalists. They also had less performance improvement over the course of their career compared to Olympic Games finalists. Kearney and Hayes [24] conducted a retrospective study of top 20 ranked senior track and field athletes in the United Kingdom and found that performance during youth had only a weak relationship with performance at senior level.

Three Norwegian brothers who have all been European 1500 m champions, were found to have had very different athletic developments [20]. The oldest (HI) took part in football, cross-country skiing and distance running during his childhood, and is ranked among the top 10 all-time best in the 1500 m in Norway in the age groups 15, 16, 17 and 18 years. The 2016 European 1500 m champion (FI), played football from the age of 7 until the age of 16 and is not among the all-time top ten in any event in any age group from 13 to 18 years. The youngest brother (JI) however, has been the best runner in Norway in all age groups from the age of 13 years. FI’s and, to some extent, HI’s development is in accordance with the findings of Anderson and Mayo [25] who reported that many elite athletes specialize late, following diversification in other sports during their youth. JI on the other hand, focused on distance running from an early age [20]. This is in accordance with the findings of a review article by Coutinho, Mesquita and Fonseca [26], who claim there is considerable evidence in the literature that both early specialization and diversified experience in other sports can lead to elite development. A study of ten elite track and field athletes from Australia, who had all finished top ten at the Olympic Games and/or World Championships, indicates that the main factors accounting for success at senior level are high self-belief, high motivation with personal goals and achievement and athletics being a central part of their lives [27].

In athletics, performance can be quantitively measured in minutes and seconds or meters and centimeters, and to qualify for an international competition such as the European Championships 2020, an athlete has to achieve the European Athletics Championship 2020—Entry Standards (EAC20ES) [28] (Table 1). In Norway, the Norwegian Athletic Federation (Norges Friidrettsforbund—NFIF) registers all-time best results for seniors, juniors and boys and girls in all track and field events from the age of 13 years. The aim of this retrospective study was to determine how many of the all-time best Norwegian track and field athletes had performed at an international level (defined as having achieved the EAC20ES) and how many of these can also be found among the 20 all-time-best results for girls and boys at the ages of 15 and 18 years. In addition, we wished to examine to what extent Norwegian track and field gold medal winners in European Championships, World Championships and Olympic Games from 1980 to 2019 are represented in the top 20 all-time best lists for girls and boys at ages 15 and 18 years.

## 2. Materials and Methods

### 2.1. Data Sample

The present study was conducted in accordance with the declaration of Helsinki. Since data in the present study are based on publicaly available resources (https://www.friidrett.no/aktivitet/statistikk), no informed consent was obtained. The study was approved by the Norwegian social science data services.

The Norwegian Athletic Federation (Norges Friidrettsforbund—NFIF) register all-time best results for women and men, for juniors and for girls and boys in all track and field events from the age of 13. The number of athletes who achieved EAC20ES in sprint, middle distance, long distance, jumping, throwing and combined events, and their best results at 15 and 18 years of age are taken from these official lists, which were updated on the 31.12.2019.

To avoid an athlete who achieved the EAC20ES in more than one event being registered multiple times, athletes were divided into the following sub-groups: sprint and hurdles; middle and long distances; jumping; throwing; combined events. Athletes who, at the age of 15 and/or 18 years, were among the top 20 in more than one event are only registered once.

Norwegian gold medal winners in European Championships, World Championships and Olympic Games from 1980 to 2019 are also from The Norwegian Athletic Federation’s (Norges Friidrettsforbund—NFIF) official statistics.

### 2.2. Different Equipment in the Throwing Events

Over the last 30 years, some changes have been made to the standards for throwing equipment in Norway in the age group of 15 years. In the hammer throw, the length of the hammer wire was 110 cm until the year 2000, after which it was increased to 119.5 cm. As such, the top 20 athletes in the age group of 15 years competing with both 110 cm and 119.5 cm hammer wire are registered. Athletes in the 15 years age group also competed with equipment of differing weights in the javelin, discus and shot-put events. The equipment weights most frequently registered for each event are included here. In shot put, this is 3.0 and 5.5 kg for girls and boys, respectively. In discus and javelin, equipment weights of 1 kg and 600 g, respectively, were used for both girls and boys. In the age group 18 years, the equipment is of the same weight and standard as that used at senior level.

### 2.3. Statistical Analyses

The number of men and woman who achieved the EAC20ES in the different subgroups, and the percentage of these who were ranked among the top 20 in the age groups 15 and 18 years, were calculated.

Descriptive statistics are presented as means and standard deviations (SD) for the age when Norwegian track and field gold medals winners in European Championships, World Championships, and Olympic Games won their first international title.

## 3. Results

Table 2 shows the total number of Norwegian athletes, and the number of male and female athletes, who have achieved the EAC20ES, and the number who have achieved the EAC20ES, in the following sub-groups: sprint and hurdle events (sprints), middle- and long-distances events (distances), jumping events (jumping), throwing events (throwing), and combined events. The number of these, and the percentage rank among the top 20 in the age groups 15 and 18 years, are also listed in Table 2.

Norwegian athletes who became Olympic Champions (OC), World Champions (WC) and European Champions (EC) from 1980 to 2019 and their best ranking in any event at ages 15 and 18 years are listed in Table 3. In addition to the track and field events, marathon is also included. International champions in cross-country and road races are not included in Table 3. The ages at which the athletes won their first international title in track and field events or marathons are also included in Table 3.

The average age at which the athletes listed in Table 3 won their first international title in track and field events or marathon was 24.7 ± 3.9 years.

## 4. Discussion

A total of 202 Norwegian track and field athletes have during all times achieved the EAC20ES (Table 1). Of these, 14.4% and 42.1% are ranked among the top 20 all-time best Norwegian athletes in one or more events at the ages of 15 and 18 years, respectively.

The data from the present study are in line with the findings of Kearney and Hayes [24] who conducted a retrospective study of the top 20 ranked senior track and field athletes in the United Kingdom. However, the percentage of top 20 ranked 15 and 18 year old boys and girls in the present study who later achieved the EAC20ES is even lower than the percentage of the top 20 ranked girls and boys at age 15 and 17 years who reached top 20 senior level in United kingdom [24]. In the study by Kearney and Hayes [24], 48% of boys and 58% of girls who were ranked top 20 at an age of 17 years were also among the top 20 ranked senior athletes. In the present study, more female athletes who achieved the EAC20ES are ranked in the top 20 at the age of 15 and 18 years than their male counterparts. This is in agreement with the findings in the study by Kearney and Hayes [24] and may be due to the fact that girls mature earlier than boys [29,30]. However, the absolute numbers of athletes having achieved the EAC20ES are in all groups, besides sprints, lower for women than for men.

The percentage of athletes ranked in the top 20 at age 15 who later went on to achieve EAC20ES is highest for jumping (31.8%), throwing (25.8%), and combined events (30%) and lowest for distance events (5.6%). For the sprint events, the percentage is 12.9%. At age 18, the percentage of top 20 athletes who later achieve EAC20ES for all events is much higher than at age 15. However, for the middle- and long-distance events, the percentage is still markedly lower than for the other events. This may be due to the fact that peak athletic performance in endurance events normally occurs at a later age than in explosive power/sprint events [31]. However, it has to be underlined that from the data in Table 1, no information can be drawn from the ranking in the lists to determine the amount of athletics training at ages 15 and 18 years, and to what extent the athletes at these ages also took part in other sports.

The percentage athletes who were ranked top 20 at the ages of 15 and 18 years is much higher for those who have won an international title: 66.7% (8 of 12 champions) in age group 15 years and 91.6% (11 of 12 champions) in age group 18 years. As such, it appears that results at a top national level at the age of 18 years may be a premise for becoming an international champion. This is in agreement with the findings of Svendsen et al. (2018), who found that the strongest predictor of senior success among Norwegian junior road cyclists was race performance at 18 years of age. However, it is in contrast to the findings of Foss, Sinex, and Chapman [1], who, after finding that World Junior Championship finalists in athletics reached their career-best performance at an earlier age and had less performance improvements during their careers than Olympic Games finalists, challenged the assertion that elite success at junior level is a prerequisite for success at senior level. It has to be underlined that, despite the fact that the Norwegian international champions listed in Table 3 showed excellent performance at the age of 18 years, only one of them (JI) won an international title at U20.

What more do we know about the 12 international athletic champions listed in Table 3? Is early specialization or early diversification the key for success as an elite senior athlete?

Among the two outstanding former world record holders and World Champions in distance running events GW and IK, we find similarities in training during their adolescence [32]. They both participated in an extensive range of sports. In addition to distance running, GW competed in sprints, jumps and throws, as well as playing handball during her early youth. IK participated in cross-country skiing and running. One difference between the two is that, up until senior level, GW did more anaerobic training than IK. The latter got her basic training through aerobics, cross-country skiing and trail running. GW achieved her best times in the 1500 m and the 3000 m. She was also best in the 800 m. IK was best over the longest distances. This might have been a consequence of differences in training during their youth and/or “genetic differences” [32]. We also know that the male distance runners included in Table 3 have taken part in a variety of sports during childhood and early adolescence. The 1996 Olympic Champion VR competed in both cross-country skiing and athletics up to the age of 16 years [33]. Of the three brothers, HI, FI, and JI, who are all European 1500 m champions, HI played football from the age of 10 to 14 years. From the age of 13 to 17 years he also competed in cross-country skiing during the winter season and in distance running during the summer season, and at the age of 17, became Norwegian junior cross-country skiing champion. FI played football from the age of ten, competed in cross-country skiing and did some track and field training. From the age of 17, he focused more and more on distance running. The youngest of the brothers, JI, started to train for athletics at the age of 7 years. He competed in sprints, hurdles, and jumps. He also competed in cross-country skiing at regional level until the age of 12. From that age he focused entirely on distance running [20].

The female and male javelin throwers in Table 3 have four and five international titles, respectively. They both showed an outstanding talent for javelin throwing from an early age and are number one and four in the all-time rankings at age 15, and both number one at age 18. However, EKH also played handball at a regional level during most of her career and after she retired as a javelin thrower.

The 400 m hurdler, twice World Champion and once European Champion, also achieved outstanding results in different track and field events from an early age. In the age group 15 years, he is ranked as the second-best long jumper in Norway of all time, and at the age of 17 years he became U18 World Champion in combined events (eight events).

Why do the international champions listed in Table 3 to a greater extent show outstanding results in their youth compared to the EAC20ES in Table 2? It is a frequent topic of discussion whether genes [34] or environment play the most important role in talent development [35]. Three of the 12 champions are brothers, which underlines the importance of favorable genes. But, in addition to genetic predisposition, these brothers are characterized by an active childhood, a gradual progression in training volume, strong family support, and mental toughness [20]. This is in agreement with Simonton [36] who argued that talent arises from dynamic processes and is a complex system beyond the environment vs. gender debate. Simonton [36] explains the development of talent from a mathematical model. In the model, different factors are weighted by importance, and include genetic dispositions, environmental (e.g., social and family support) and development constraints (e.g., structure of training and competition program). Simonton’s model is in accordance with Mallett and Harrahan [27] who found that elite track and field athletes have, (1) high motivation with achievement and personal goals, (2) high self-belief, and (3) athletics being a central part of their lives.

## 5. Conclusions

In Norway, a total of 202 track and field athletes achieved the European Athletics Championship 2020—Entry Standards (EAC20ES) in one or more events during all times. Of these, 14.4% are ranked among the all-time top 20 athletes in Norway in one or more events in the age group 15 years, and 42.1% in the age group 18 years. However, there is no available information regarding the amount of athletics training at ages 15 and 18 years, or to what extent the athletes at these ages also took part in other sports.

During the last four decades, 12 Norwegian athletes have won one or more international titles in track and field events or marathons. Of these champions, the number who were ranked among the top 20 in the age groups 15 and 18 years is much higher: 66.7% and 91.6%, respectively. This could indicate that those who go on to become international champions perform at a higher level in adolescence than other athletes who also reach senior international level. However, due to the low number of international champions, the date should be interpreted with caution.

## Figures and Tables

**Table 1:** European Athletics Championships 2020—Entry Standards (https://www.european-athletics.org).

Men	Discipline	Women
10.28	100 m	11.44
20.80	200 m	23.35
46.40	400 m	52.65
1:47.30	800 m	2:02.50
3:39.50	1500 m	4:11.00
13:44.00	5000 m	15:50.00
28:50.00	10,000 m	33:20.00
8:45.00	3000 m steeplechase	9:55.00
13.90	110 m hurdles/100 m hurdles	13:30
50.70	400 m hurdles	57.95
NES	Half-Marathon	NES
2.24	High Jump	1.90
5.60	Pole Vault	4.45
7.95	Long Jump	6.60
16.60	Triple jump	13.90
20.00	Shot Put	17.00
63.50	Discus	57.00
74.45	Hammer	69.00
80.50	Javelin	58.00
7850	Combined Events	5850

NES = No entry standard.

**Table 2:** The number of Norwegian athletes who achieved the EAC20ES in sprints, distances, jumping, throwing and combined events, and the number and percentage (%) of these ranked among the top 20 in the age groups 15 and 18 years.

Group	Number of Norwegian Athletes Who Achieved EACS20ES			Number and Percentage (%) Ranked in the Top 20 Age 15			Number and Percentage (%) Ranked in the Top 20 Age 18		
	Total	Men	Women	Total	Boys	Girls	Total	Boys	Girls
sprints	31	15	16	4 (12.9%)	1 (6.7%)	3 (18.8%)	20 (64.5%)	9 (60.0%)	11 (68.8%)
distances	108	74	34	6 (5.6%)	4 (5.4%)	3 (8.8%)	24 (22.0%)	17 (22.9%)	7 (20.5%)
jumping	22	12	10	7 (31.8%)	4 (5.5%)	3 (30%)	16 (72.7%)	10 (83.4%)	6 (50%)
throwing	31	24	7	8 (25.8%)	5 (20.8%)	3 (42.9%)	21 (67.7%)	16 (66.7%)	5 (71.4%)
combined events	10	7	3	3 (30.0%)	2 (28.6%)	1 (33.34%)	4 (40.0%)	2 (28.6%)	2 (66.7%)
total	202	132	70	29 (14.4%)	16 (12.1%)	13 (18.6%)	85 (42.1%)	54 (40.9%)	31 (44.3%)

**Table 3:** Norwegian athletes who have become OC, WC and/or EC from 1980 to 2019, their best ranking in any event at ages 15 and 18 years, and the age at which they won their first international title in track and field events or marathon. If their best ranking at ages 15 and 18 years is in another event than the one they have won an international title in, the event is noted in parentheses. M = male, F = female.

Athlete	Event	Year	OC	WC	EC	15 Years	18 Years	Age
GW (F)	marathon	1983		X			3 (1500 m)	30 **
IK (F)	10.000 m	1986, 1987		X	X	2 (1500 m)	*	30
EKH (F)	javelin	1993, 1997, 1994, 2000	X	XX	X	1	1	27
GM (M)	200 m	1994			X		16 (100 m)	25
SH (M)	High jump	1994			X	16	2	23
VR (M)	800 m	1996	X				5	24
HH (F)	high jump	1997		X		16	1	30
AT (M)	javelin	2004, 2008, 2006, 2010, 2009	XX	X	XX	4	1	24
HI (M)	1500 m	2012			X	9	2	21
FI (M)	1500 m	2016			X		14 (800 m)	23
JI (M)	1500 m, 5000 m	2018			X	1	1	18
KW (M)	400 m hurdles	2017, 2019, 2018		XX	X	2 (long jump)	1 (400 m)	21

* IK did not compete in athletics at the age of 18, but instead competed as an international cross-country skier at junior level. ** GW was also 5 times World Cross-Country Champion; the first time was in 1978 at the age of 25 years.
